# Molecular and Functional Characterization of *Bacopa monniera*: A Retrospective Review

**DOI:** 10.1155/2015/945217

**Published:** 2015-08-27

**Authors:** Koilmani Emmanuvel Rajan, Jayakumar Preethi, Hemant K. Singh

**Affiliations:** ^1^Department of Animal Science, School of Life Sciences, Bharathidasan University, Tiruchirappalli 620024, India; ^2^Laboratories for CNS Disorder, Learning & Memory, Division of Pharmacology, Central Drug Research Institute, Lucknow 226001, India

## Abstract

Over the last 50 years, laboratories around the world analyzed the pharmacological effect of *Bacopa monniera* extract in different dimensions, especially as a nerve tonic and memory enhancer. Studies in animal model evidenced that *Bacopa* treatment can attenuate dementia and enhances memory. Further, they demonstrate that *Bacopa* primarily either acts via antioxidant mechanism (i.e., neuroprotection) or alters different neurotransmitters (serotonin (5-hydroxytryptamine, 5-HT), dopamine (DA), acetylcholine (ACh), *γ*-aminobutyric acid (GABA)) to execute the pharmacological effect. Among them, 5-HT has been shown to fine tune the neural plasticity, which is a substrate for memory formation. This review focuses on the studies which trace the effect of *Bacopa* treatment on serotonergic system and 5-HT mediated key molecular changes that are associated with memory formation.

## 1. Introduction

*Bacopa monniera* (L.) Wettst., which belongs to the family Scrophulariaceae, is an annual creeping plant found in wet, damp, and marshy areas. The leaves and stem of the plant are used for medicinal purposes traditionally [1]. In the ancient Indian system of medicine, namely, Ayurveda,* B. monniera* known as “Bhrami” has been classified under Medhya Rasayana and described in ancient ayurvedic medical encyclopedias, namely,* Charaka Samhita*,* Sushrutha Samhita,* and* Astanga Hrdaya,* as cure for mental disorders and loss of intellect and memory. It has been tested in different animal models to understand its effect on memory [2, 3] and antiamnesic activity [4–9]. These pharmacological properties lead to clinical trial of* B. monniera* extract in elderly persons to improve cognitive performance and memory [10–15]. In parallel,* Bacopa* is a main constituent in the preparation of ayurvedic medicine prescribed for cognitive dysfunction. In addition, several research groups and pharmaceutical companies formulated* Bacopa* for clinical use in different countries including India, New Zealand, Australia, and United States of America. Earlier, many reviews have discussed pharmacological property of* B. monniera *in a broad perspective; however, no comprehensive article has yet shown its effect on molecular level. In this review, we summarize the* in vivo* experiments that suggest that* B. monniera* treatment enhances cognitive function by altering the molecular targets through serotonergic system.

## 2. Bioactive Compounds in* B. monniera* Leaf Extract

Series of biochemical studies identified different pharmacological compounds from ethanolic extracts of* Bacopa*, which include alkaloids (brahmine, nicotine, and herpestine), saponins (monnierin, hersaponin), sterols (b-sitosterol, stigma-sterol), d-mannitol, acid A, and betulinic acid [16–18]. The principal constituents of* B. monniera* are triterpene saponins of the dammarane class, which have been named bacosides and bacopasaponins. There are two types of saponins, jujubogenin and pseudojujubogenin, which differ only in the nature of the sugar units in the glycosidic chain and the position of the olefinic side chain in the aglycone. These saponins are complex mixture of closely related structures, namely, bacosides A_1_ [19] and A_3_ [20] and bacopasaponins A–G [21–23]_._ Two new dammarane-type jujubogenin bisdesmosides, bacopasaponins E and F [24], pseudojujubogenin glycosides, bacopasides I and II [25], phenylethanoid glycosides, namely, monnierasides I–III with the known analogue plantainoside B [26], and bacopasides III, IV, and V [27] have also been identified. The major chemical entity shown responsible for neuropharmacological effects of* B. monniera* is bacoside A (64.28%) and bacoside B (27.11%); the latter differs only in optical rotation. The bacoside A (bacogenins A1, A2, A3, and A4) derives from two triterpenoid saponins: pseudojujubogenin and jujubogenin on acid hydrolysis [16–18, 28]. All these bacogenins (especially A4) are rich in the standardized extract of* Bacopa* which is termed as bacosides-enriched standardized extract of* Bacopa* (BESEB CDRI-08) that contains 55 ± 5% bacosides (Lumen Marketing Company, Chennai, India), and BESEB CDRI-08 is mentioned as BME in this paper.

## 3. Neuropharmacological Activity of BME

### 3.1. Learning and Memory

*Bacopa* treatment has been reported to improve behavior of different laboratory animal models under variety of experimental conditions. Oral administration of BME improved spatial learning of rats and mice in Morris water maze [4, 5, 29–31]. Interestingly, several other studies demonstrated that it also improved spatial working memory in different mazes like plus maze [32, 33], Y-maze [34, 35], radial arm maze [34, 36], Barnes maze [36], T-maze [37], Hole board [35], and modified Y maze [38]. In addition, it also improved negative reinforcement (foot-shock motivated brightness discrimination task, conditioned avoidance response) and positive reinforcement (conditioned taste aversion) based memory [2, 39]. Similarly, in passive avoidance task and fear conditioning task* Bacopa* treatment increased the transfer latency and freezing response [33, 35, 37, 38, 40–42], whereas, in contextual cues associated with odor, BME treated rats showed less latency to retrieve the reward [43] and exhibited improved discrimination of novel object [38, 44, 45]. In addition, it has been stated that* Bacopa* treatment induced dendritic arborization of neurons in hippocampal and basolateral amygdala [46, 47], which possibly enhanced neural plasticity.

## 4. *B. monniera* Extract Treatment Ameliorates Chemicals Induced Dementia

Interestingly, several studies investigated the pharmacological effect of BME against different chemical compounds that induce anterograde/retrograde amnesia by targeting different neuronal system. These studies reported that BME effectively attenuated anterograde/retrograde amnesia induced by chemical compounds such as scopolamine, an acetylcholine receptor antagonist [2, 6, 7, 22, 36, 40, 48, 49], diazepam, a positive allosteric modulators of *γ*-aminobutyric acid (GABA) type A receptor [4], N_*ω*_-nitro-l-arginine (L-NNA), a nitric oxide synthase inhibitor [8, 9], BN52021, a receptor antagonist for platelet activating factor [48], and sodium nitrite, a anticholinergic drug [48]. In addition, memory impairments caused by Okadaic acid, a selective inhibitor of protein phosphatase [31], aluminium-chloride which causes oxidative damage [50], autistic symptoms induced by sodium valproate, a weak blocker of sodium ion channels, and inhibitor of GABA transaminase [51] were also ameliorated by* Bacopa* treatment.

## 5. Uptake of Bacosides

We have learned from pioneering works about different active compounds in* B. monniera* extract [16–18]. As a first step to validate the effect of BME on the reported behavioral improvements, Charles et al. [35] confirmed that orally treated BME was uptaken into the system. HPLC analysis showed the presence of bioactive compound bacoside A in the serum of BME treated rats. The bioactive compounds in the BME could directly or indirectly interact with neurotransmitter systems to enhance learning and memory. Since the bacosides present in the BME are nonpolar glycosides [25–27], they can cross the blood-brain barrier (BBB) by simple lipid-mediated passive diffusion [52], and its bioavailability in brain has been confirmed by the biodistribution of radiopharmaceuticals [53] effectively activating the cascade which participates in the memory enhancing mechanism.

## 6. Activation of Neurotransmitter Systems by Bacoside

**Figure 1:**
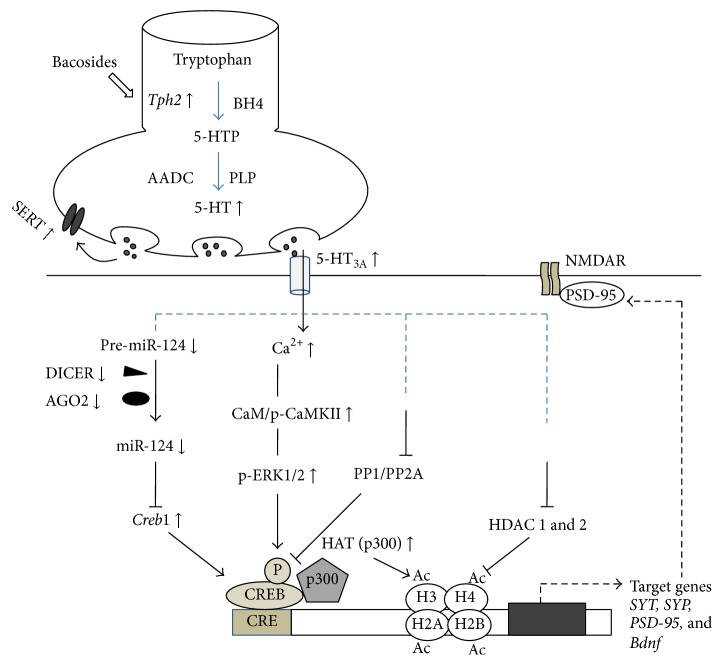
Diagram showing the possible mechanism of serotonin mediated signaling pathway activated by BME during learning. (↑: increase; ↓: decrease).

The balanced functions of various neurotransmitters such as acetylcholine (ACh) [2, 40], serotonin (5-hydroxytryptamine, 5-HT) [2, 54], catecholamine [55], *γ*-aminobutyric acid (GABA) [56], and glutamate (Glu) [8] were all altered by BME treatment. It has been reported that the BME treatment increased the 5-HT level in the hippocampus, hypothalamus, and cerebral cortex [54], and also modified the ACh concentration directly/indirectly through other neurotransmitter systems. As a first step, Rajan et al. [41] estimated the level of neurotransmitters to understand the effect of BME treatment. They found that BME treatment during postnatal period significantly upregulated the level of 5-HT, ACh, GABA, and Glu. In contrast, it reduced the level of dopamine (DA). Notably, the reported inhibitory effects of cholinesterase activity of BME may possibly increase the level of ACh and enhance memory [33, 40]. On the other hand, 5-HT receptors present in the GABAergic neuron [57] may activate the GABAergic neurons [58, 59], which enhances the release of GABA. In fact, increased GABA level in hippocampus could activate the inhibitory GABA receptors on cholinergic system that leads to inhibition of ACh release [60, 61], but 5-HT receptors may directly act on the cholinergic system and increase release of Ach [62]. These proceedings and the observed trend in the 5-HT level have drawn the attention to analyse the effect of BME on 5-HT system. Further, studies were designed to test the pathway associated with 5-HT system (Figure 1). Observed effect of BME on neurotransmitter systems and the molecules involved in the signaling pathway are shown in Table 1.

## 7. BME Treatment Regulates the Synthesis of Serotonin

Earlier studies demonstrated that increasing level of tryptophan hydroxylase (TPH) mRNA expression elevated TPH activity and 5-HT metabolism, which profoundly could influence the synaptic 5-HT activity [63, 64]. Further, serotonin transporter (SERT) is known to critically uptake the 5-HT by transport across presynaptic membrane [65]. The upregulated level of 5-HT by BME raises the question, does it alter the level of TPH2 and SERT? Interestingly, Charles et al. [35] showed that TPH2, SERT mRNA expression was upregulated and the level persisted even a week after the BME treatment [35]. The upregulated SERT expression could regulate the reuptake of released 5-HT and control the duration and intensity of serotonergic activity at the synapse. This could be one of the mechanisms that enhance the learning and memory processing and it fits well into established concept in different models [66, 67]. In addition to these studies,* in silico* analysis suggested that interaction of bacosides (A, A_3_) with TPH2 possibly alters the activity of TPH2 that could be one of the mechanisms for increased 5-HT synthesis [68].

## 8. Activation of 5-HT Receptor by BME Treatment

Previously, it has been found that synaptically released 5-HT exerts its function through their diverse receptors [69]. Activated receptors either positively or negatively regulate the downstream signaling cascade that is involved in regulation of synaptic plasticity [70–72]. In view of these reports, expression of 5-HT receptors (5-HT_1A_, 5-HT_2A_, 5-HT_4_, 5-HT_5A_, 5-HT_6_, and 5-HT_7_) after BME treatment was examined. Notably, 5-HT_3A_ receptor expression was increased compared to all other receptors. It is the only metabotropic receptor, and its expression could be stimulated by endogenous 5-HT which may facilitate the hippocampal-dependent task [73, 74]. Hence, the role of 5-HT_3A_ in hippocampal-dependent learning could be tested by using 5-HT_3_ antagonist 1-(*m*-chlorophenyl)-biguanide (*m*CPBG), which effectively impairs the retention of the conditioned response [75] in both short- and long-term memories [76]. The 5-HT_3_ antagonist* m*CPBG has facilitated gaining insight into the BME induced 5-HT_3A_ receptor mediated role in hippocampal-dependent learning and its regulation of other neurotransmitters. Interestingly, treatment of BME ameliorated the antagonistic effect of* m*CPBG. The combination of* m*CPBG and BME treatment recorded improvement in behavioural task accompanying the upregulation of 5-HT_3A_ receptor. Considering the interaction of multiple neurotransmitters involved in learning and memory network [77–80], it could be interesting to know the interaction of 5-HT_3_ receptor in activation/inhibition of other neurotransmitter systems.

The upregulated 5-HT_3A_ receptor might regulate serotonergic system and may interact with other neurotransmitters that are involved in learning and memory [58, 67, 81]. It should be noted that 5-HT_3A_ is a heteroreceptor; its stimulation by means of* m*CPBG has been reported to enhance GABA and DA levels and inhibit the release of ACh [74]. The activation of 5-HT_3_ receptors in dopaminergic neuron could facilitate the release of DA [82, 83], and* m*CPBG inhibits dopamine uptake by binding with dopamine transporter [84], thereby increasing the synaptic dopamine level. On the other hand, the anticholinesterase activity of BME [40] and other regulatory mechanisms of BME are also involved in the regulation of ACh level and memory enhancement [33, 85].

A noteworthy point is that it did not alter the level of Glu. This suggests that glutamate neurons in the hippocampus may not colocalize with 5-HT_3A_ receptor [59]. The observed changes are indication of the facilitatory effect of BME on long-term and intermediate forms of memory through 5-HT_3A_ receptor.

## 9. Activation of Protein Kinases-CREB Pathway

A pioneering study in 1976 described that serotonin stimulation increases the level of cyclic adenosine monophosphate (cAMP) by the adenyl cyclase in the neuronal cells [86]. Subsequent study by Castellucci et al. [87] established that activation of cAMP mediates downstream signaling process through phosphorylating proteins, namely, cAMP-dependent protein kinase or protein kinase A (PKA). Upon activation, cAMP-dependent PKA dissociates into regulatory and catalytic subunits. The catalytic subunit of PKA drives to activate mitogen activated protein kinase (MAPK)/extracellular signal-regulated kinase (ERK1/2) [88, 89]. It has been shown that activation of protein kinases (MAPK/ERK) can induce the phosphorylation of the key transcription factor CREB, which is a positive regulator of memory consolidation [90–93]. These proceedings triggered us to test whether the BME treatment induced activation of 5-HT_3A_ receptor regulated synaptic plasticity through protein kinase and cAMP response element binding (CREB) protein signaling pathway. It is noteworthy to mention that treatment of BME increased the phosphorylation of ERK1/2 and provides a physiological and functional meaning for the observed different forms of memory [42]. If the p-ERK activity is decreased/increased, one would expect concomitant changes in the CREB and CREB targeted gene expression and functional consequences [94–97]. It should be noted that the induction of p-CREB1 is involved in the regulation of synaptic proteins synthesis, which are known to be involved in synaptic plasticity related events in hippocampus [98] and their synthesis is necessary for the consolidation of long-term memory (LTM) [99–102]. Preethi et al. [39] found that level of both total and phosphorylated CREB protein was increased in the BME treated individuals. When BME treated before* m*-CPBG treatment, the* m*CPBG mediated suppression of CREB phosphorylation was attenuated by BME, thus adding additional support to the effect of BME in regulation of PKA-CREB pathway.

## 10. Activation of CREB Regulation through MicroRNA-124 by BME

Long-term memory formation requires synthesis of new proteins [103, 104], which is regulated by mRNA transport and translation [105]. At this point, several studies proposed that microRNAs (miRNAs) are one of the factors that regulate expression of gene [106, 107] which could be regulated by level of miRNA/biosynthesis of miRNA. There are two molecules, Dicer and Ago2, involved in the regulation of miRNA biosynthesis [108]. It is noteworthy to mention that there is an interaction between miR-124 and 5-HT, because the stimulation of the latter has been shown to downregulate the expression of miR-124 during 5-HT-induced synaptic facilitation [109]. Thus, we thought that BME treatment might alter the level of miR-124 expression and the molecules involved in its biosynthesis pathway. Subsequently, we found that BME treatment reduced the level of Dicer, Ago2 mRNA, and protein [39]. Reduction in Dicer has been known to enhance synaptic plasticity [110]; the formation of miRNA-induced silencing complex (miRISC) requires the activation of Ago2 [111]. Further, this study revealed that reduction of Dicer and Ago2 directly downregulated miR-124 level in BME treated individuals. Conversely, inhibition of 5-HT activity by treating with* m*CPBG showed upregulated Dicer, Ago2, and miR-124 [39]. It has been postulated that the downregulation of miR-124 would lead to the upregulation of CREB [109]. Though it is well established that 5-HT can upregulate* Creb1* mRNA level [112], recent studies claimed that miR-124 might directly bind to* Creb1* 3′UTR and regulates the expression of CREB [109, 113]. Indeed, upregulated CREB reciprocally regulates the miRNA [109, 114]. This in turn regulates the activation of immediate early genes that ultimately facilitates synaptic plasticity [115–118]. These cellular events demonstrate that BME possibly regulates the transcriptional regulators to fine tune transcription factors.

## 11. Phosphorylation of CREB Regulated by BME Treatment

Contrary to the protein kinases, protein phosphatases (PPs) act as dephosphorylating enzymes that dephosphorylate the molecules like CREB [119]. PPs critically regulate the phosphorylation events that favor forgetting [120], cognitive decline in ageing [121, 122], and suppress learning and memory. In brain, several PPs are known to be expressed. Among them, Ser/Thr phosphatases (PP1, PP2) are the most likely candidates that negatively act on the phosphorylation of CREB [123–125] and thereby downregulate the transcription of CREB targeted genes [120, 126, 127]. BME treatment significantly reduced the PP1*α* and PP2A level in hippocampus, which appears to be responsible for observed BME mediated enhanced memory [42]. This study revealed the contribution of BME in regulation of CREB phosphorylation that favors the transcription of CREB targeted genes to memory formation. Moreover, it supported the earlier reports which showed inhibition of PPs to enhance memory formation [120, 124, 125, 128–130], but the exact mechanism that inhibits PPs is not yet studied.

## 12. Chromatin Modifications Differentially Regulated by BME Treatment

Studies in memory highlighted chromatin alteration and epigenetic changes that are associated with CREB activation. Contribution of histone tail acetylation and deacetylation in chromatin are widely known to be involved in the formation of long-term memory and synaptic changes [131–133]. Histone deacetylase (HDAC) inhibitors are known to induce acetylation of histones (H3, H4). It has been reported that HDAC inhibitors repress the HDAC-PP1 complex and thus block dephosphorylation of CREB [134, 135]. On the other hand,* in vitro* and* in vivo* studies claimed that transcriptional induction of CREB occurred by pSer133, which requires histone acetylase (HAT)—CREB binding protein (CBP/p300) [136, 137]. P300 contains intrinsic HAT activity and it has been shown to interact with CREB [138–140]. Manipulation in p300 leads to reduction in the histone acetylation and impairs hippocampus dependent memory [141–143]. These reports prompted us to examine the potential role of BME in chromatin modifications especially with histone acetylation and deacetylation.

An earlier study reported significant enhancement of p300 level in hippocampus of BME treated groups, but not in control groups after training [42]. These reports suggest that BME plays an agonistic role for p300 in hippocampus; further it may acetylate H3 and H4 histones [144–146]. Accordingly, we found that BME treatment induced marked increment in the level of Ac-H3 and Ac-H4 in hippocampus [42]. These results agree with the earlier studies, in which HDAC inhibitors have been found to induce acetylation of histones (H3, H4) and improve memory [147–150]. In addition, the level of HDAC 1 and HDAC 2 in the hippocampus of BME treated group was decreased compared to control group. The reduction in HDAC 1 and HDAC 2 levels together with increased acetylation of histones in BME groups added additional evidence to the mechanism of BME [42].

## 13. BME Treatment Activates the Synaptic Proteins to Induce Synaptic Plasticity

Behavioural response to the stimuli is basic functional circuit formation between the neuronal cells. The molecular mechanism underlying the circuit (synaptic plasticity) is likely to provide insight to role of molecules/molecular complexes. The communications between the neuronal cells are initiated by the recruitment of adhesion molecules in pre-post synaptic neurons [151, 152]. Synaptic plasticity depends on activity strength, which leads to release of neurotransmitters to the synaptic cleft. However, the release of neurotransmitters is critically regulated by synaptic proteins synaptotagmin-I (SYT-1) and synaptophysin (SYP). SYT-1 is sensitive to Ca^2+^ and conserved at least in vertebrates [153]. This synaptic vesicle protein is exclusively involved in synaptic vesicle docking and regulating release of neurotransmitter [154]. Another key synaptic protein SYP is playing important role in regulation of synaptic vesicle association by protein-protein interactions [153]. It is a vesicle-associated regulatory protein which is involved in plasticity related changes in the hippocampus [155, 156]. The levels of SYT-1 and SYP were upregulated after BME treatment which possibly established the synaptic communication and synaptic function [43]. BME treatment upregulated the synaptic proteins (SYT-1, SYP), which is possibly by the elevated level of 5-HT. The level of signaling components is essential for neurotransmission and synaptic plasticity. The upregulated synaptic proteins could enhance neurotransmission and synaptic plasticity. However, this should be transferred to postsynaptic neurons. There are two key postsynaptic proteins (post synaptic density protein 95 (PSD-95) and Ca^2+^/calmodulin dependent protein kinase II (CaMKII)) distributed densely. Acute phosphorylation and localization of PSD-95 and CaMKII is fundamental to synaptic function [157]. They are critical for long-term potentiation (LTP) and information storage [158, 159]. Translocation of CaMKII to postsynaptic region by autophosphorylation is necessary for early phase of memory formation, where it controls the phosphorylation of different postsynaptic proteins [160]. The induction and phosphorylation of CaMKII depends on the release of 5-HT [161]. Genetic manipulation and pharmacological studies pointed out the critical role of CaMKII in synaptic plasticity and memory formation [161, 162]. BME treatment upregulated the induction and phosphorylation of CaMKII; it could be by the level of 5-HT, thus the improved memory recorded. PSD-95 is a core component in the architecture of synapses [163, 164] involved in localization of receptors, clustering of synaptic signaling proteins, and synapse stabilisation [164–166]. The level of PSD-95 increases at synapses during learning/learning-induced plasticity [167, 168]. Earlier, we demonstrated that PSD-95 was upregulated after BME treatment [43]; upregulated PSD-95 may increase the interaction between PSD proteins and enhances synaptic transmission [169–172]. These results suggest that BME treatment activates the synaptic proteins; thus neurotransmission and synaptic plasticity are enhanced between the neurons.

## 14. Conclusion

Taken together, bacosides present in the* Bacopa* extract has been known to improve cognitive function by modulating different neurotransmitters. However, this review focused on the studies which provide much attention to the serotonergic system, in which, starting from* in silico* approach to alternation in 5-HT levels, their receptors and associated signaling cascades known to be involved in synaptic plasticity and memory enhancement were discussed. These studies provide molecular evidence to possible mechanism of BME on serotonergic system and its associated pathway.

## Figures and Tables

**Table 1:** Summary of *Bacopa monniera* treatment effects on serotonergic system and its associated pathway.

Neurotransmitters	Effects	Genes (mRNA)	Effects	Genes (Protein)	Effects	References
Serotonin	↑	*Tph2 *	↑			[35]
		*SERT *	↑			

Serotonin Dopamine Acetylcholine GABA Glutamate	↑ ↓ ↑ ↑ ↑	*5-HT1*_*A*_	—			
		*5-HT2*_*A*_	↑			
		*5-HT3*_*A*_	↑			
		*5-HT4 *	—			[41]
		*5-HT5 *	—			
		*5-HT6 *	—			
		*5-HT7*	↓			

		*Nrf2*	↑	SYP SYT t-*α*CaMKII p-*α*CaMKII PSD-95	↑ ↑ ↑ ↑ ↑	[43]

		*Dicer* *Ago2 * *miR-124* *Creb1*	↓ ↓ ↓ ↑	DICER AGO2 t-CREB1/2 p-CREB1/2	↓ ↓ ↑ ↑	[39]

		*Bdnf* *PP1α*	↑ ↓	t-ERK1/2	↑	
				p-ERK1/2	↑	
				t-CREB1/2	↑	
				p-CREB1/2	↑	
				Ac-H3	↑	[42]
				Ac-H4	↑	
				HDAC1	↓	
				HDAC2	↓	
				p300	↑	
				PP2A	↓	

↓: decrease; ↑: increase.

## Abbreviations

5-HT: 5-Hydroxytryptamine
ACh: Acetylcholine
BESEB CDRI-08: Bacosides-enriched standardized extract of* Bacopa*
CaMKII: Ca^2+^/calmodulin dependent protein kinase II
cAMP: Cyclic adenosine monophosphate
CBP: CREB binding protein
CREB: Cyclic adenosine monophosphate (cAMP) response element binding
DA: Dopamine
ERK1/2: Extracellular signal-regulated kinase
GABA: *γ*-Amino butyric acid
Glu: Glutamate
HAT: Histone acetylase
HDAC: Histone deacetylase
L-NNA: N_*ω*_-nitro-l-arginine
LTM: Long-term memory
LTP: Long-term potentiation
MAPK: Mitogen activated protein kinase
*m*CPBG: 1-(*m*-Chlorophenyl)-biguanide
miRISC: miRNA-induced silencing complex
miRNAs: MicroRNAs
PKA: Protein kinase A
PPs: Protein phosphatases
PSD-95: Postsynaptic density protein 95
SERT: Serotonin transporter
SYP: Synaptophysin
SYT1: Synaptotagmin I
TPH: Tryptophan hydroxylase.
